# Perceptions of national guidelines and their (non) implementation in mental healthcare: a deductive and inductive content analysis

**DOI:** 10.1186/s13012-015-0234-0

**Published:** 2015-04-01

**Authors:** Boel Sandström, Ania Willman, Bengt Svensson, Gunilla Borglin

**Affiliations:** Department of Health, Blekinge Institute of Technology, SE-371 79 Karlskrona, Sweden; Department of Health Sciences, University of Lund, SE-221 00 Lund, Sweden; Blekinge Center of Competence, SE-371 81 Karlskrona, Sweden; Department of Care Science, Malmö University, SE-205 06 Malmö, Sweden

**Keywords:** Evidence-based practice, Guidelines, Implementation, Mental health, Nursing, PARIHS, Qualitative research

## Abstract

**Background:**

National guidelines are being produced at an increasing rate, and politicians and managers are expected to promote these guidelines and their implementation in clinical work. However, research seldom deals with how decision-makers can perceive these guidelines or their challenges in a cultural context. Therefore, the aim of this study was twofold: to investigate how well Promoting Action on Research Implementation in Health Services (PARIHS) reflected the empirical reality of mental healthcare and to gain an extended understanding of the perceptions of decision-makers operating within this context, in regard to the implementation of evidence-based guidelines.

**Methods:**

The study took place in the southeast of Sweden and employed a qualitative design. The data were collected through 23 interviews with politicians and managers working either in the county council or in the municipalities. The transcribed text was analysed iteratively and in two distinct phases, first deductively and second inductively by means of qualitative content analysis.

**Results:**

Our deductive analysis showed that the text strongly reflected two out of three categorisation matrices, i.e. evidence and context representing the PARIHS framework. However, the key element of facilitation was poorly mirrored in the text. Results from the inductive analysis can be seen in light of the main category *sitting on the fence*; thus, the informants’ perceptions reflected ambivalence and contradiction. This was illustrated by conflicting views and differences in culture and ideology, a feeling of security in tradition, a certain amount of resistance to change and a lack of role clarity and clear directions. Together, our two analyses provide a rich description of an organisational culture that is highly unlikely to facilitate the implementation of the national guidelines, together with a distrust of the source behind such guidelines, which stands in stark contrast to the high confidence in the knowledge of experienced people in authority within the organisational context.

**Conclusions:**

Our findings have highlighted that, regardless of by whom guidelines are released, they are not likely to be utilised or implemented if those who are responsible for implementing them do not trust the source. This aspect (i.e. contextual trust) is not covered by PARIHS.

## Introduction

Internationally and nationally evidence-based guidelines, which aim to secure and improve care for clients, patients and/or service users, are being produced and released at an increasingly rapid pace. Still, with a few exceptions in the Scandinavian countries, guidelines are usually passively distributed, and very few are accompanied by instructions or government support regarding how they are to be implemented and, thereby, to change healthcare practice. The purpose of guidelines is to direct politicians and managers in their decision-making regarding the planning of care, the prioritisation of treatments to offer and the allocation of resources [1]. The task of rolling out guidelines in an organisation also falls on this level and is, therefore, the decision-makers’ responsibility. The intention is for the recommendations in the guidelines to be used by staff in order to ensure good quality and safe care for patients. However, if those who make decisions (i.e. politicians and managers) are to promote the guidelines as intended, they should also be disseminated with respect to this specific target group, while taking into account the cultural context. In order to accomplish this, more knowledge about how decision-makers can perceive implementation processes and guidelines is required. This knowledge is important, since it is well known that implementation (i.e. ‘the systematic uptake of clinical research findings and other evidence-based practices into routine practice’) [2] is a slow, costly and cumbersome process. This is shown, not least, by the vast number of studies that exist concerning barriers to research utilisation [3] and difficulties in implementing evidence-based practice (EBP), as well as studies discouraging the use of ineffective interventions [4]. At the same time, there is currently a great deal of knowledge on which strategies can be effective in implementation, as several systematic reviews of interventions for the implementation of new knowledge have been published [5-8]. Lectures by outside instructors, reminders, multi-faceted interventions and workshops have proved to be effective interventions, as have computerised decision support and rewards. Most results, however, come from studies in the medical field and cannot always be immediately transferred to other contexts or professionals [9,10]. Differences in education, professional roles, responsibilities, work and decision-making may also require completely different strategies [11,12]. Knowledge of the specific cultural context in which an implementation is supposed to take place is, therefore, crucial to success [13-15].

Mental health appears to be a particularly tricky culture to manage, especially when it comes to implementation of EBP [14,16,17]. In the mental healthcare field, the concept of EBP has generated considerable controversy and been much debated [16,18]. Critics of EBP question, among other things, the high value placed on evidence from randomised controlled trials (RCTs) and argue that a quantitative, positivistic approach could be difficult to apply to the complex and messy mental healthcare context [18]. The adoption of guidelines within mental healthcare is also known to be tardy [17]. Sceptics argue that guidelines may standardise the care for specific individuals in particular contexts and that an over-reliance on the scientific can lead to a reduction of the humanities [18]. Instead, professional knowledge and experience together with the relationship between the therapist and patient have been emphasised.

One reason for this study was the recent act by the Swedish National Board of Health and Welfare (NBHW) in March 2011 to publish guidelines for psychosocial interventions in healthcare and social services [1], which were specifically designed to address people with schizophrenia or schizophrenia-type conditions (hereinafter, ‘guidelines for schizophrenia’). People with schizophrenia and other serious mental disabilities often need comprehensive and long-term support from their communities in a range of life areas [1]. Even so, these people are not routinely offered care or social support based on evidence. Responsibility for the provision of such support lies with politicians and managers, who determine what care and support should be offered and provide the funding and resources to realise it. Consequently, a good understanding of the perceptions of those who are in charge of making guidelines a reality in clinical practice (i.e. politicians and managers) is essential to overcoming potential context-specific barriers to the implementation and use of guidelines.

One way to approach this issue and to increase the understanding of where and what things, in the chain of events, might go wrong is from a theoretical point of view. This is especially true because theory can facilitate an understanding of the complexity of the many factors at various levels that influence the process of implementing EBP guidelines [19-21]. Factors known to influence the implementation process are associated with the organisational context (cf. [22]), the characteristics of the person adopting the guidelines (cf. [23-25]) and the characteristics of the evidence [26]. There is a vast range of theories, frameworks and models describing these factors, which are available to use in the process of implementing new knowledge and change within the healthcare field [26-29]. Still, several of these are incomplete, insufficiently tested and based on organisational theories for which the micro perspective is not investigated. Moreover, the need and usefulness of theory has been questioned in the past [30,31]; however, there seems to be growing support for its value [32-34], as well as a general cry for the increased use of theory in research. Still, there remains a lack of research on implementation process models that are tangible and easy to translate when faced with the complex task of implementing evidence in practice. One way to proceed is to use and test the existing frameworks.

To investigate how well an existing framework reflects the empirical reality of a mental healthcare context, we decided to test the Promoting Action on Research Implementation in Health Services (PARIHS) framework. This framework was developed to facilitate the understanding of the complexities involved in successful implementation, and it is often used in nursing. The implementation of research in practice is seen as a process involving the interplay of the key elements evidence, context and facilitation. Evidence consists of research evidence, knowledge from clinical experience, patients and carers’ experience and local context information. Context refers to the environment or setting in which the proposed change is to be implemented and includes the sub-elements culture, leadership and evaluation. Facilitation refers to the process of enabling the implementation of evidence into practice. The three elements with their sub-elements can be mapped on to a high-low continuum. Successful implementation (SI) of evidence-based knowledge benefits from evidence that is scientifically sustainable and consistent with the professional consensus and patient experiences, i.e. “high” evidence. The organisation should have clear leadership, a culture characterized by willingness to change and systems for monitoring and feedback, i.e. “high” context. Finally, there need to be experienced people who can facilitate change, i.e. “high” facilitation. Least SI occurs when context and facilitation are insufficient. Poor contexts can be overcome by suitable facilitation, and the chances of SI are still feeble even in a satisfactory context, if the facilitation is. PARIHS was inductively developed by Kitson and colleagues [35] and has been subsequently refined over time [21,36,37]. PARIHS has good face and content validity. Still, the framework needs to be tested further in different contexts [26]. The Swedish Society of Nursing has translated PARIHS and introduced it in professional journals for Swedish nurses. Despite this, the framework seems sparingly used in planning for implementation efforts in Swedish healthcare. The core concepts of PARIHS are designed to cover the elements that need attention in the implementation process and should, therefore, be reflected in discussions of clinical practice. Consequently, the aim of this study was twofold: to investigate how well PARIHS reflected the empirical reality of mental healthcare and to gain an extended understanding of the perceptions of decision-makers operating within this context, in regard to the implementation of evidence-based guidelines.

## Method

This study employed an explorative (i.e. introductory and illuminating) qualitative design [38]. This type of research begins with a phenomenon of interest and the intent to explore the full nature of that phenomenon. Thus, data were collected through semi-structured interviews (ibid.), and the transcribed texts were analysed iteratively in two distinct phases—by deductive and inductive qualitative content analysis.

### Study context

In Sweden, healthcare is publicly funded. Society’s responsibility for providing good health and social care to people with mental illnesses and mental disabilities is divided among the state, county councils and municipalities. The county councils’ obligation lies in offering treatment, while the municipalities’ main responsibility is social services [39]. Regionally and locally, elected politicians of county and municipal councils are in charge of finances and, consequently, of setting budgets for mental health services run by top-level mental healthcare managers. The present study took place in one county consisting of five municipalities in the southeast parts of Sweden. About 150,000 people populate the five municipalities in the area. The county-based psychiatric resources in each municipality consist of an outpatient clinic and a mental healthcare clinic, where patients can go several times a week for individual or group treatments. There are approximately 45 inpatient beds in the county. The county’s municipalities share a psychiatric emergency room, a dependency and abuse clinic, an eating disorder unit, a sexologist and a cognitive clinic with a senior team. Each municipality also has a certain number of places available in sheltered housing care and provides occupational and job training.

### Sample

The sample consisted of 18 mental healthcare managers and 5 politicians (*n* = 23). A two-staged purposive sampling technique was used [38]. This technique allows a researcher to sample respondents while using his/her knowledge about the target population (ibid.). In this case, the sample targeted all politicians on both the regional and the local level, as well as mental healthcare managers and top-level and middle managers whom we knew had participated in a seminar about the provisional guidelines for schizophrenia in May 2010. The participants were, therefore, presumed to have perceptions regarding the guidelines and their implementation in clinical practice. The seminar was initiated by the NBHW with the purpose of encouraging decision-makers (i.e. managers and politicians) to begin to support the process of implementing the guidelines in their clinical practice areas. Thus, in stage 1, all eligible respondents from the seminar (30 mental healthcare managers and 18 county council politicians; *n* = 48) were purposively targeted via email and informed about the study. One week after the email, the first author (BoS) contacted all 48 by phone and asked about their interest in participating. Fifteen agreed to participate (managers *n* = 12, politicians *n* = 3). Of the rest of the eligible respondents, nine had finished their assignment and/or position, seven declined to participate and seventeen could not be reached by phone, despite several attempts.

In stage 2, we purposively targeted [38] additional healthcare managers and politicians who had not attended the NBHW seminar but who also had responsibility for mental healthcare in the municipality and county council. Recruitment in stage 2 aimed at ensuring varied and rich information and at making sure the sample did not become too homogenous (i.e. influenced by the attended seminar). The first author (BoS) took the opportunity, during the phone calls, to ask the seven eligible respondents in stage 1 who had declined to participate whether they could suggest a replacement, and a further eight respondents (managers *n* = 6, politicians *n* = 2) were recruited through this snowball sampling strategy (ibid.). All respondents signed a written informed consent and brought it along to the interview.

The final sample (*n* = 23) consisted of 16 women and 7 men. Of these, 14 were employees in municipal services, and 9 were employed in county-based activities. Their ages ranged between 25 and 67 years (median = 52 years; mean = 52 years). Thirteen respondents had a university education, five held a diploma in nursing or social work (of these, four also had a specialist education) and five had a secondary school education. Their time in their current positions ranged from 4 months to 30 years (median = 7 years; mean = 7 years).

### Data collection

The semi-structured interviews [38] were conducted from April to June 2012. The interviews began with an overarching question that sought to encourage narration: ‘Can you please tell me your thoughts regarding the released national guidelines?’ A similarly phrased question was used to cover their views regarding the implementation: ‘Can you please tell me your thoughts regarding the implementation of the guidelines?’ General probing techniques, such as ‘How do you mean?’ and ‘Please tell me more’, were used [38]. The interviews lasted between 40 and 80 min and were conducted at a time and place chosen by the informants. The interviews were recorded, saved as audio files and transcribed verbatim in their entirety.

### Data analysis

Qualitative content analysis was used, inspired by the method descriptions, primarily by Elo and Kyngäs [40] and secondly by Hsieh and Shannon [41]. The data analysis was conducted in two distinct phases: deductive and inductive. This methodological integrative and iterative approach has so far not been extensively described in the nursing literature. However, some more recent papers [42,43] have described content analysis procedures using theory as a grid for analysing textual data. One of the major benefits of content analysis is its flexibility in terms of research design and that the use of deductive and/or inductive ways should be determined by the purpose of the research [40]. Here, the study purpose is twofold: to investigate how well PARIHS reflected the empirical reality of mental healthcare and to gain an extended understanding of the perceptions of decision-makers operating within this context, in regard to the implementation of evidence-based guidelines. Deductive content analysis is useful when *a priori* theory (here PARISH) exists about a phenomenon [41]; thus, the structure of the content analysis is operationalised on that previous knowledge. Deductive content analysis is also especially useful in cases of retesting data in a new context such as here in the field of mental health (cf. [40]). The inductive approach is recommended when there is insufficient or fragmented knowledge about the phenomenon [40].

#### The deductive content analysis

In the first phase, the transcribed texts were deductively analysed while applying the PARIHS framework [44] as a lens. The analysis process started with several readings of the written material to become familiar with it. The transcribed interviews were read and reviewed by the first author (BoS) to gain an overall impression and sense of the texts. Simultaneously, two of the co-authors (BeS, AW) individually read a number (*n* = 8) of randomly chosen interviews whereas one of the co-authors (GB) read all of them (*n* = 23), also to get an overall understanding of the material. Then, in the first phase, a structured categorisation matrix [40] was developed for each one of the PARISH frameworks’ three key elements and their individually belonging sub-elements. These matrices were also structured in a way that allowed the text to be assessed as representing *high* or *low*. Each one of the structured categorisation matrices was used as a lens when the data again was read through. The interview texts were then reviewed for content and coded for correspondence with the key elements and their individually belonging sub-elements in the structured matrices. Only aspects from the data that fit the matrices were chosen [39], and after being assessed as representing *high* or *low*, coded text was transferred into its corresponding position in the matching matrix (Table 1). In order to facilitate the encoding phase, diagnostic questions corresponding to PARIHS and developed by Kitson and colleagues [37] were used. Text that was undecided or judged as not fitting in any of the structured matrices was saved in a separate document together with the first author’s (BoS) written rationale for her assessment [40]. When this was done for all text, the co-authors (BeS, AW, GB) appraised the three matrices to ensure an agreement between the first author’s assessments of *high* and *low*, and of the positions in the matrices. Now the team also appraised the document containing text that was initially assessed as either undecided or as not fitting in any of the matrices. This appraisal meant that the non-fitting text could be excluded as the team judged it to not concern the study aim, e.g. text excluded did not narrate about guidelines or implementation. Whereas, the undecided text could, now in the light of the complete analysis, be transferred into its corresponding position in the matching structured categorisation matrix. Thus, no text was left behind in the first phase of the analysis.

**Table 1:** **Overview of the deductive analysis process**

**Statements high**	**Statements low**	**Evidence**
‘…it’s a support for me in my work.’ (131)	‘So we need, after all…you’re supposed to not slavishly…but we may be working with other methods that work, just…give as good an effect, exactly, so you cannot just buy it. I mean, it’s not that…oh, it’s great with evidence-based methods, but you still have to be vigilant.’ (130)	Research
‘I think it should be…it should lead Swedish health care; it should be scientific, but then, where there is no science, but we still see through our experience that it makes a difference; we must still retain it. But, at the same time, when we have evidence, we will use it, and if we don’t have it, we have to use our professional judgment.’ (137)	‘Some people might have an incredible ability to create supporting relationships. And some people may try their entire career, and it is no one who feels that they have a supporting relationship with them anyway.’ (136)	Clinical experience
‘…The patient is the one who knows if this is good or not; it is not the therapist, it’s not the professional…because then it will have no effect on the patient. I can sit and think a lot, but if the patient don’t think it’s good. …to constantly question, are we working on the right things, is this…does this work for you, or should we do otherwise…’ (143)	‘But how much it has spread and how pervasive it has been out there among the consumers is difficult to know.’ (114)	Patients experience
		Local information
		**Context**
‘…but now…yes, we are in a transformation phase now, where we have to take resources from employment and have more people working with IPS. It is our goal and our vision today.’ (119)	‘…So it is well to sit down and have time to plan what we are going to do. That might not really always…everyone just runs on, I feel sometimes, and I’m one of those who are running. I think we get very stressed by it [the release of the guidelines], and really, I wonder how much…how much it comes out the other end, but we are in a wheel.’ (114)	Culture
‘…this should we focus on, and we‘ll offer it in the whole county, so it is a management decision in the top-level psychiatric management team, but the discussions are, of course, out in practice.’ (132)	‘Then there is the top-level manager who is in charge. It is the person and what he believes. Yes, in this case, H now then…’ (135)	Leadership
	‘To me, it’s so important to follow up on how things are that they call back somehow. That one…this when you are told that this far have we come today, to update people on how far we are…this has happened since then. For, otherwise, it is just a paper product again. And I’m a bit like that…I can miss this…we are so bad at follow up.’ (121)	Evaluation

Numbers in parentheses refer to the informant.

*IPS* Individual Placement Support.

#### Inductive content analysis

In the second phase, all the text was subjected to an inductive qualitative content analysis inspired by Elo and Kyngäs’ description [40]. This was conducted in order to gain an extended understanding, i.e. an understanding going beyond the earlier categorisation of the texts. The analytic process also focused on the latent content which involves an interpretation of the underlying meaning mediated by the text [45]. The analysis started with the text being read again with the aim to get a sense of its meaning outside the grouping in the matrices, and open coded (i.e. notes and possible headings are written in the text while reading it). The text was then, guided by the codes describing aspects of the content, structured into sub-categories, based on their commonalities. Thereafter, the text within each sub-category was analysed to identify variations, similarities and differences. Sub-categories were then named using words that characterised their content. Finally, the sub-categories were read once again, compared and grouped into main categories based on their belonging (Table 2). The research team independently read and analysed the text and met regularly to discuss and reach a consensus in the different steps of the analysis. To further ensure the trustworthiness of the analysis, quotes from the informants are reported in the results.

**Table 2:** **Overview of the inductive analysis process**

**Quotations**	**Sub-categories**	**Generic categories**	**Main category**
‘…we have had a lot of discussions of the method itself, and where did they [NBHW] come up with this, and what is the evidence that this particular method has any effect?’ (127)	A bone of contention	A running battle	
‘Yes, I think it was very clear that different perspectives existed and that things can be viewed from different perspectives. I always get that perception that the country council work in one way and the municipality work in a complete different way. It is like two different worlds are meeting.’ (129)	A world apart		
‘…I think one is stuck in very old experience and routine that feels safe for them. Letting go and learning something new is not always that easy.’ (138)	Ingrained in the walls	Better safe than sorry	Sitting on the fence
‘…but when we sat in the workgroup I thought that…because everything is so very evidence-based today, it is supposed to be so much evidence, and I have been working in psychiatry for a while and seen the pendulum swing from the right to the left and so on, from different trendy…trends that have come and gone.’ (135)	The resistance		
‘I am sure it is our responsibility to roll it out in some way, but how that will work in practice and which ones who would be receptive for the information and understand it, and how to make it understandable when one self has quite a lot of difficulties to get a grip of it…that is the question.’ (113)	Passing the buck	A fragmented approach	
‘Okay, what should we take away if we decide to squeeze this in; what should we take away when we already have a full organisation?’ (121)	Mixed messages		

Numbers in parentheses refer to the informant.

### Ethical considerations

According to the Swedish Ethical Review Act Involving Humans (SFS [46]:460), this study did not need ethical clearance by a regional ethical review board. Even though this means being granted an exemption from requiring ethics approval, the study was conducted in strict compliance with the established ethical guidelines of the Declaration of Helsinki [47]. Thus, the study was conducted within an appropriate ethical framework. Consequently, all informants received both oral and written information regarding the study’s aim, confidentiality issues and their rights to—at any point in time during their participation—withdraw from the study without leaving any explanation. All participants additionally handed in a signed form of informed consent. Data was stored securely and anonymous in compliance with the Data Protection Act [48].

## Results

The use of the PARIHS framework as a lens made it possible to investigate whether the transcribed texts would fit into the theoretical content of the key elements: evidence, context and facilitation (Table 1). The deductive analysis showed that the text strongly reflected two of the three categorisation matrices, i.e. evidence and context representing the PARIHS framework [21,36,37,44]. The deductive analyses are reported in keeping with the three key elements in the PARIHS framework.

### The key element evidence and its belonging sub-elements

The key element evidence is, in the PARIHS framework, regarded in a broad sense in order to include the sub-elements of *research*, *clinical experience*, *patients experience* and *local information* [21,36,37,44].

The sub-element *research* (i.e. the evidence base underpinning the guidelines) was represented by texts reflecting informants who valued the guidelines and believed in the evidence base underpinning them, as well as texts reflecting informants who expressed scepticism and questioned the evidence base. The latter represented the *low* end of the continuum, and the informants’ uncertainty about how the guidelines were conceived, their mistrust in the quality of the evidence supporting the guidelines and their doubt concerning whether implementing the guidelines would make any difference were all represented here. In contrast, at the *high* end of the continuum, statements about the relevance of the guidelines, their trustworthiness, how they made research accessible and how working in accordance with them would be cost effective and result in human paybacks were reflected (Table 1).

The deductive analysis also mirrored statements reflecting the high value placed on professional knowledge, which signified the sub-element *clinical experience*. Statements presenting clinical experience as a set of old habits, routines or traditions or as an uncritical and unreflective belief in one’s own experience represented the *low* end of the continuum. At the *high* end of the continuum, statements concerning the importance of combining research evidence with professional judgement in decision-making about care, learning from one another and of reflecting on ones’ own experience were discernible (Table 1).

The sub-element *patient experience* was denoted by statements reflecting the service users as passive participants/recipients (i.e. as not being involved in the process of implementing the guidelines, despite viewing them as important parts of the decision-making process). This represented the *low* end of the continuum, in which the fact that very few service users had been informed about the existence or content of the guidelines and the prevalence of doubts about the service users’ ability to comprehend this type of information were implied. At the *high* end of the continuum, statements about the importance of the individuals’ involvement in all decisions regarding their lives and the significance of regular meetings between service user associations and management with regard to mental healthcare were represented (Table 1).

The analysis reflected that the sub-element *local information* (i.e. audit, performance and quality improvement) was poorly mirrored. The few statements that reflected the fact that systematically collected local information were obtained all concerned lab tests and consumer surveys in the social services, which were executed over sparse intervals and were, therefore, not within the remit of this study’s aim (Table 1).

### The key element context and its belonging sub-elements

Statements reflecting uncertainty over whether existing policy documents supporting EBP were known represented the sub-element culture and whether individuals understood their meaning at all levels in the organisation. The text also mirrored a fear that knowledge about the significance of EBP was similarly scarce. The implementation of the guidelines was perceived to be a cumbersome and demanding process, in which time and resources were limited and the workload was heavy. Very few of the managers had informed their staff of the guidelines or of the strategies for implementing parts of the guidelines, and, as a result, the staff lacked an awareness of the connection between the education they had received targeting the methods recommended in the guidelines and the guidelines themselves. Statements also illustrated trust in local authorities, an unwillingness to change and a dislike of government directives. These statements signified the *low* end of the continuum. In contrast, statements reflecting clarity regarding values and beliefs and an appreciation of staff and consumers represented the *high* end. Here, managers in the county council and municipalities worked in close collaboration, and all staff members had received education in two of the recommended methods in the guidelines (Table 1).

The sub-element *leadership* was characterised by statements reflecting traditional leadership, in the sense that decisions were made inside the management group. The leadership was also decentralised in the sense that the managers had the potential to act quite freely when making decisions about the practices they managed. A few statements were supportive of a transformational leadership style, in which the manager encourages and empowers his staff. At the *low* end of the continuum, statements mirroring the lack of support and clear directives from the political body denoted the sub-element, together with concerns about the fact that responsibility and authority are left to the managers without control or requirements for evaluation. On the *high* end of the continuum, statements reflected good collaboration among managers, a united strategy and role clarity (Table 1).

The sub-element *evaluation*, in the sense of systematic procedures for evaluation or feedback, was not mirrored in the text; however, statements reflected that both evaluation and feedback were valued and desired (Table 1).

### The key element facilitation

Facilitation involves an individual with certain skills and attributes in carrying out a specific role. A facilitator’s mission is to help individuals, teams and organisations apply evidence-based knowledge to everyday practice [44,49]. None of the statements reflected facilitation as described within the framework. Several statements, however, revealed the opinion that an individual fulfilling such a role was needed (Table 1).

### Results from the inductive analysis

The objective of the inductive analysis was to gain a sense of possible explanations of the text outside its grouping in the matrices. The results revealed that the informants’ views could be understood through the perspectives of three generic categories—*a running battle*, *better safe than sorry* and *a fragmented approach*—along with their underpinning sub-categories. Together, these outlined the basis for the main category *sitting on the fence* (Table 2). The numbers in brackets refer to the informants.

### A running battle

Within the category *a running battle*, the sub-category *a bone of contention* was interpreted as highlighting the often conflicting views held regarding the guidelines. The sub-category *a world apart* reflected disparities in culture and ideological outlook but also attempts to overcome differences. Overall, the category revealed a range of different and often opposing views concerning the guidelines and their implementation in clinical practice.

#### A bone of contention

The sub-category *a bone of contention* reflected different perceptions regarding the guidelines that were interpreted as *a bone of contention.* Some informants saw the guidelines, as complementing existing knowledge and practices, providing direction and making the mission clear. It was considered important that the methods chosen for implementation were evidence based and exerted with fidelity. There were also concerns that, without guidelines, there was a risk that personal ideological beliefs would guide mental healthcare; in contrast, working in accordance with guidelines was considered a possible path towards fairer, safer and better-quality care for service users.

I think it’s really important that we have these guidelines, it should not be any difference where we live in the country what we can offer the target group, and we must have a code of rules. (131)

Other informants expressed clear scepticism towards the guidelines and EBP in general, which was regarded as being just a buzzword. The sub-category also mirrored a distrust expressed by some of the politicians, as well as some of the managers, towards the source behind the guidelines (i.e. the NBHW). There were also questions about how the guidelines had been selected and why the guidelines resulted in these particular ones. The guidelines were perceived to have been compiled by experts without any knowledge or roots in everyday clinical practice. The guidelines were also regarded as temporary—something that could be changed or withdrawn. Furthermore, the sub-category was denoted by criticism regarding the applicability and usefulness of the guidelines.

#### Worlds apart

The sub-category *worlds apart* reflected views concerning difficulties in collaborating on the implementation of the guidelines due to differences in cultures, ideological positions and approaches. Differences were interpreted to exist between public authorities, such as the county council, and the municipalities, as well as between influential individuals. Cultural differences were also acknowledged between units, which were also said to have different degrees of willingness to change. The view that leading experts or authorities affected the care of and the range of treatments available to the target group was put forward.

…The medical profession had different perceptions regarding treatment. And that is not unusual but…it felt as if it was very…some were traditionalists, so what you always have done was the right way to do, you should not try something new. Because it wouldn’t work. And then there were the proponents of new methods and among these was the debate… (129)

Concern was raised regarding the possibility that an individual physician’s personal preferences would have a greater impact than the guidelines on the care offered. Some of the middle managers expressed perceptions of being not completely autonomous in their profession. Although the intention of the manager was to ensure care in accordance with the guidelines, dependence on the physician or on higher-level managers sometimes made this impossible. The personal views of authorities could even influence other cultural contexts through collaboration across agency boundaries. Although cultural differences between the county council and the municipalities were acknowledged, collaboration was perceived by the top managers as being important means for facilitating future cooperation.

### Better safe than sorry

The category *better safe than sorry* encompassed the sub-categories *ingrained in the walls* and *the resistance*, reflecting both the security of the old and well known, which was captured in statements like, ‘it’s hard to turn on certain things that are culture’, and a certain amount of fear concerning change and ‘the new’.

#### Ingrained in the walls

The sub-category *ingrained in the walls* meant that there were norms and traditions that seemed difficult to change. This sub-category expressed that it was better to stick to old and familiar ways because it was not certain that new methods or practices would lead to better care. The culture was characterised by a common history and a strong consensus, but also by an element of struggle. Views that the culture of mental health was special and different from that of somatic healthcare were reflected, along with a sense of invisibility in comparison.

…mental health can often not be seen, so then it is also silent with regard to the large somatic care… (124).

The notion that change takes time in mental health was interpreted as a part of the culture, as a tradition of staff members choosing to do things their own way and as a mixing of different methods. A strong belief in experiential knowledge, which was not subjected to critical scrutiny, could also be discerned in the statements. People in authority who had extensive experience were bestowed high statuses and were rarely questioned.

#### The resistance

In the sub-category *the resistance*, the idea of implementing the guidelines was mirrored as being challenging for those who had different ideological approaches than what was promoted in the guidelines. Individual backgrounds, education and personal values were interpreted as possible causes for resistance, as was a fear of not being able to use the skills and tools that staff and therapists had acquired through education and experience. Some therapists felt that their experience and knowledge were being questioned. Perceptions of the guidelines as a threat to skilled staff, who were successful without endorsing EBP, could also be discerned. Furthermore, a lack of awareness of the release of the guidelines, as well as knowledge and misconceptions about their content, was also interpreted as contributing to resistance on the parts of both staff and several of the managers. This sub-category reflected perceptions of the guidelines as a passing trend that many did not bother to acknowledge:

…it doesn’t matter if we have some damn guidelines; no one is following them anyway. People are running their own races. (135)

The value inherent in providing a variety of different methods of treatment instead of following the recommendations in the guidelines became discernible, along with strong personal beliefs that it is the encounters with the patient and the subsequent relationship that is the crucial prerequisite to good-quality mental healthcare.

### A fragmented approach

In the category *a fragmented approach*, the sub-category *passing the buck* was interpreted to reflect views concerning the responsibility for disseminating information to the staff about guidelines. The sub-category *mixed messages* was interpreted as mirroring perceptions of conflicting expectations and demands, as well as a lack of feedback and follow-up.

#### Passing the buck

The sub-category *passing the buck* was interpreted to reflect an inconsistency between the acknowledgement of responsibility for the implementation of guidelines, but a failure to take on the duty or enact a plan for realising this task. The decisions about what care should be offered to service users were a concern for top-level managers and politicians, and this hierarchical structure was interpreted to be clear-cut. The way in which the actual implementation should be promoted was, however, not clearly stated. This was captured in statements like

I am sure it is our responsibility to roll it out in some way, but how that will work in practice and which ones who would be receptive for the information and understand it, and how to make it understandable when one self has quite a lot of difficulties to get a grip of it, that is the question. (113)

The importance of being a role model and of enthusing and arousing an interest in guidelines among mental healthcare staff was also highlighted by the managers, as was the importance of informing staff about guidelines. Still, none of this was prioritised, and the reasons given included a lack of time and a lack of resources. Feelings of stress, requirements of keeping costs down and tough priority choices concerning the needs of different diagnostic groups were also mentioned as things that took precedence over the task of rolling out guidelines. The sub-category *passing the buck* became especially visible in different ad hoc and split views concerning the ways in which implementation of the guidelines could be promoted, preferably with the help of a facilitator of some kind. It was suggested that a compilation of verbal and written information could be helpful, as could education, a conference and the spreading of good practical examples. Furthermore, someone should be specially appointed to disseminate information about guidelines, policies and important news from state agencies, as this dissemination was perceived as something that managers could not possibly keep up.

#### Mixed messages

Ambiguity surrounding the requirements and expectations of managers regarding the process of implementing the guidelines became discernible in the sub-category *mixed messages*. On one hand, the importance of clear direction from politicians with regard to what to expect from managers and practitioners was emphasised. Adherence to the recommended interventions in the guidelines was, according to the politicians, not optional and the responsibility of the politicians and the NBHW to monitor and evaluate the implementation process of national guidelines. On the other hand, the politicians’ desire not to interfere with matters of everyday mental healthcare practice was highlighted, as was their confidence in the managers’ knowing best regarding patient care.

When we have the meeting of the Board, there is always a manager present. And when issues come up then it’s the manager who is knowledgeable of the guidelines simply speaking, so that is what one’s trying to follow. (142)

The interpretation of the statements reflected differing views regarding whether EBP was a goal or not. There were those who argued that it was written into the county council’s policy document that care should be evidence based, while others argued that such a goal did not exist. It also became clear that the economy or county council’s financial budget had priority over practice and resources; moreover, efforts towards quality improvements, such as implementing guidelines, were very limited.

## Discussion

This study aimed to investigate how well the PARIHS framework could reflect the empirical reality of a mental healthcare context and explain the perceptions of managers and politicians operating in that context with regard to national guidelines for mental health and their implementation. The results of our deductive analysis imply that, although the semi-structured interviews were not guided by key elements in PARIHS [44] and, instead, sought to examine perceptions of the guidelines and their implementation more generally, the text still fit well with two of the three matrices, i.e. evidence and context (Table 1). Results from the inductive analysis can be viewed in light of the main category *sitting on the fence*; thus, informants’ perceptions reflected ambivalence and contradiction. This was illustrated by conflicting views and by differences in culture and ideology (*a running battle*), a feeling of security in tradition and a certain amount of resistance to change (*better safe than sorry*) and a lack of role clarity and clear direction (*a fragmented approach*) (Table 2).

One noteworthy result revealed in our inductive analysis was a clear distrust of the source (i.e. the NBHW) behind the guidelines. The group of people putting together the guidelines were perceived as academics—that is, they were perceived as existing far from everyday clinical practice and lacking in personal experience from the field. The concept of trust has internationally been researched within fields such as management, sociology, social psychology and occupational psychology [50]. Commitment to change and innovation implementation behaviour has for example been explored in relation to employees’ trust in top management [51]. Research within the field of mental health [52] and within primary care has more extensively explored trust between patients and clinicians [53]. Consequently, trusting the messenger as part of change and/or of implementing guidelines has shown to be of importance between organisations and its individuals as well as between the individuals within the organisation. Thus, it seems reasonable to propose that the same trust needs to exist between the governmental agencies realising national guidelines, healthcare organisations and the individuals within the context to initiate change and implement EBP.

Lakeman [14] suggests that mental health fields should be viewed as ‘cultures of care’, in which culture and caring practices are embedded in localised sub-cultures, each with their own norms, traditions and rituals. Therefore, understanding how mental healthcare professions and practices change and why requires a similar understanding of the cultural context. Here, a strong confidence in the knowledge of experienced people with high status in the organisation was expressed. This result is supported by the conceptual model, put forth by Szulanski [54], of sticky knowledge, in which a lack of credibility in the source may lead to an impaired transfer of new knowledge. A trust’s status and trustworthiness, on the other hand, may positively influence the process. Szulanski’s [54] suggestion that credibility in some organisations may be linked more with time served and with loyalty to the prevailing order than to the implementation of innovations also supports our analysis. A high level of trust for people with extensive experience and the sense of security possible through a common history and shared values and beliefs were expressed by the informants. It is known that nurses tend to rely more on knowledge from their colleagues and personal experience than on formal knowledge sources [55,56]. Our results indicate that the decision-makers appear to do the same. In PARIHS, evidence is one of the key concepts and other authors highlight the credibility of the scientists who developed the evidence as conclusive, assuming that strong evidence is so convincing that it is not questioned [57,58]. However, it became obvious in this study that it is not sufficiently convincing for evidence to be scientifically robust; the evidence must also come from a source that is considered reliable.

Culture cannot be ignored in the attempt to successfully implement guidelines. It is known that the implementation of EBP is promoted in cultures where research is valued, but not in cultures that emphasise tradition and rituals. It is also likely that a culture in which experiential knowledge is more trusted than knowledge based on research will need substantial time to transform into a culture that is more open to change [59]. Mental health has been described as suffering from conservative and backward-locking working methods [60] and as a culture in which particular value is placed on experiential and tacit knowledge [61]. This supports our two analyses, which together offers a description of an organisational culture that is unlikely to facilitate the implementation of guidelines. The results from the deductive analysis revealed that knowledge derived from experience was highly valued, while perceptions regarding the value of research-based knowledge (e.g. guidelines) varied considerably. This picture was further clarified through the inductive analysis, in which the sub-category *ingrained in the walls* was interpreted to represent strong tradition, belief in experiential knowledge and an acceptance that change occurs slowly in mental healthcare. The importance of gaining an understanding of cultural context before any implementation or change of practice has been emphasised in existing research [13-15]. Still, the concept of culture continues to be elusive and is often referred to as ‘the black box of practice’ [62]. There is a great deal of research about the concept of culture, but the literature is broad, and the conceptions are diverse [26,63]. Culture is said to include a variety of phenomena, such as norms, traditions and rituals [14], and to influence the ways in which things are done, understood, judged and valued [13]. It is clear, however, that the concept needs to be better defined and split into additional parts. Only then will it be possible to describe and understand what, in the cultural context, needs to be addressed before the implementation of new knowledge or any change of practice.

In our deductive analysis, the lack of perceptions regarding the key element of facilitation became evident. By facilitation, Harvey et al. [49] meant an individual particularly appointed to carry out the role of a facilitator and, thereby, to support people in changing their practices through helping and enabling them. However, the results of the inductive analysis revealed that the tasks of adapting the guidelines and planning for their implementation had to be performed by the managers, with varying outcomes. Efforts to implement the guidelines were seen by the managers as time consuming and difficult to enact. The daily struggle to work within a tight budget to make ends meet with the available resources had to come first. This was supported by previous research, in which the lack of time and a heavy workload were associated with unsuccessful implementation [64,65]. Appropriate facilitation, on the other hand, has been found to overcome poor contexts (e.g. [66,67]) and to be pivotal in moving evidence into practice [44,49]. If the process of implementing evidence is to be successful, it might be that guidelines must be followed by a call to local governments to appoint facilitators to support the implementation.

### Study limitations

The sampling was purposively done and emanated from the participant list from a seminar regarding guidelines for schizophrenia. Significantly more managers (*n* = 30) than politicians (*n* = 18) participated in the seminar. The response rates from eligible participants were fairly low and biased towards managers, with low representation of politicians. However, it is common that only about half of those who are asked agree to participate, and managers as well as politicians are busy people. We therefore believe that the sample reflects reality as it is. Qualitative content analysis can be evaluated for trustworthiness by means of its credibility, dependability, conformability and transferability [68]. This study was based on 23 interviews, and the sampling was purposively conducted in order to make the results dependable. Content saturation was achieved after twenty interviews, although another three were conducted in order to confirm the results. There were some difficulties in mapping the findings onto *high* or *low*, and first impressions could, in some cases, be misleading. Examples include statements that, at first, seemed to be expressions of clear values and beliefs, which should be situated towards *high* on the continuum. However, the interpretation revealed that some of these values and beliefs were not necessarily in line with what was recommended in the guidelines and could, instead, hinder the process of implementing them. The same applied to statements that seemed to reflect a transformational leadership, but for which the objective was not necessarily to adopt or enforce the use of guidelines. There is always more than one probable interpretation of a text; however, in this case, all of the authors worked together throughout the analysis. Interpretations and categorisations were compared and discussed, and the resultant findings represent the most credible understanding of the narratives. By describing the analytical procedures used and by presenting quotations from the interview texts, we hope to ensure trustworthiness and dependable findings. A qualitative study like this one is limited with regard to its transferability and its relevance to other types of settings. However, we believe that the knowledge obtained from this study could be useful in planning the implementation of guidelines or EBPs in similar contexts, as well as in future implementation efforts within mental healthcare.

## Conclusions and relevance to practice

Our findings have highlighted that, regardless of from whom guidelines are released, they are not likely to be utilised or implemented in the care of patients if those further down in the hierarchy do not trust the source, in this case the NBHW where the latter can be said to be equivalent with for example the UK National Institute of Clinical Excellence. The importance to trust the messenger on different levels is supported by several others [50-53] as well as by Szulanski’s [54] model, which illustrates the relationship between the trust and credibility of a source. However, our review of the literature implies that more health service research is needed exploring the impact of trust between agencies and receivers for the implementation of national guidelines. The deductive analysis implies a dislike of government directives and a tendency to trust local authoritarians. The inductive analysis provided an even clearer picture, in which the developers of guidelines were perceived as researchers without knowledge of everyday clinical practice. Instead, the informants expressed a strong belief in people with authority and extensive experience within an organisation. This finding was also supported by the conceptual model of knowledge stickiness [54], in which credibility in certain organisations is suggested to be linked with status and time served and could, therefore, be damaging to the implementation process. However, this important aspect (i.e. contextual trust) is not covered by PARIHS. Thus, though national guidelines might not be instantly welcomed by politicians and managers, there is reason to believe that the process will become sticky. Therefore, the process must move from a passive diffusion to a more active implementation, in which each link in the chain—from governments, politicians and managers at the local level to the staff that is expected to use the knowledge—must take responsibility.
